# A new unmanned aerial vehicle intrusion detection method based on belief rule base with evidential reasoning

**DOI:** 10.1016/j.heliyon.2022.e10481

**Published:** 2022-09-05

**Authors:** Yawen Xie, Wei He, Hailong Zhu, Ruohan Yang, Quanqi Mu

**Affiliations:** a School of Computer Science and Information Engineering, Harbin Normal University, Harbin 150025, China; b Rocket Force University of Engineering, Xi'an 710025, China; c Northwestern Polytechnical University, Xi'an 710072, China

**Keywords:** UAV intrusion detection, Interpretable model, Evidential reasoning, Belief rule base, Global optimization

## Abstract

With the growing security demands in the public, civil and military fields, unmanned aerial vehicle (UAV) intrusion detection has attracted increasing attention. In view of the shortcomings of the current UAV intrusion detection model using Wi-Fi data traffic in terms of detection accuracy, sample size reduction, and model interpretability, this paper proposes a new detection algorithm for UAV intrusion. This paper presents an interpretable intrusion detection model for UAVs based on the belief rule base (BRB). BRB can effectively use various types of information to establish any nonlinear relationship between the model input and output. It can model and simulate any nonlinear model and optimize the model parameters. However, the rule combination explosion problem is encountered in BRB if there are too many attributes. Therefore, an evidential reasoning (ER) algorithm is proposed for solving this problem. By combining the capabilities of the ER and the BRB methodologies, a new evaluation model, named the EBRB-based model, is proposed here for predicting UAV intrusion detection, even in the case of a massive number of attributes. The global optimization of the model is ensured. A new interpretable and globally optimized UAV intrusion detection model is proposed, which is the main contribution of this paper. An experimental case is used to demonstrate the implementation and application of the proposed UAV intrusion detection method.

## 1 Introduction

With the growing demand for higher privacy protection and safety in the public, civil and military fields, unmanned aerial vehicle (UAV) intrusion detection has attracted extensive attention all over the world in the past decade [1], [2]. In January 2015, a micro-UAV crashed on the lawn of the White House, which triggered concerns about safety measures [3]. Due to UAV interference, airport runways were closed three times in 2016 [4]. Therefore, detecting UAV intrusion safely and effectively is essential.

Many scholars have put forward various solutions. Birnbach et al. [5] proposed a method for the intrusion detection of UAVs based on the received strength of Wi-Fi signals. This method can use cheap COTS hardware to detect invading UAVs with minimal preconfiguration. It is based on the available measurement data of most systems supporting Wi-Fi and provides a wide range of deployment options. Sciancalepore et al. [6] proposed using network traffic identification to detect the statuses of UAVs flying or lying on the ground, which proved that network traffic classification can be effectively used to detect the statuses of UAVs. The classification algorithm tree-j48, random forests, and neural networks were applied to various UAV traffic datasets to identify the statuses of UAVs. The results show that a strong guarantee and a very short delay were realized. Bisio et al. [7] proposed a UAV detection method based on Wi-Fi statistical fingerprint analysis. Four machine learning algorithms, namely, random trees, random forests, sequence minimum optimization, and logical regression, were used to detect data traffic. The results showed that the method could effectively detect the presence of unauthorized UAVs. The effectiveness of this method was due to an increasing number of commercial UAVs using Wi-Fi for control and FPV video streaming protocols to drive. Alipour Fanid et al. [8] proposed a machine learning method for UAV detection and operating mode identification based on encrypted Wi-Fi traffic. This method extracts the main features from the packet size and packet arrival interval, then considers the measurement times of different features in the training phase, adopts weighted single norm regularization, and combines the optimization of feature collection and output into a single objective function. To address the fuzziness of the arrival time of data packets when calculating the cost function, they used maximum likelihood estimation to find the arrival times of incoming data packets, collected a large amount of Wi-Fi traffic from eight forms of UAVs, and comprehensively evaluated the proposed methods. The experimental results show that these methods can identify UAVs within 0.15-0.35 s, with an accuracy of 85.7-95.2%. In the LOS and NLOS links, the UAV detection ranges were 70 meters and 40 meters, respectively. Support vector machine and random forest classification algorithms were used to detect the UAV operating mode, and the results show that they effectively identified UAVs. Sciancalepore et al. [9] proposed a method for detecting the presence of remotely controlled UAVs in multiple heterogeneous environments. By analyzing traffic characteristics such as packet arrival time and size, the standard random forest classification algorithm was applied to eavesdrop on traffic. The experiment proved that the presence of UAVs in a variety of heterogeneous scenarios could be effectively identified.

Researchers have made significant progress in UAV intrusion detection. However, the following problems remain. First, the evaluation accuracy of the above model is often unsatisfactory. The reason for the low accuracy is the large parameter setting error and the lack of a suitable optimization mechanism. Second, in typical data-driven models, each model has multiple nodes with multiple layers. Thus, many parameters must be estimated. This requires a lot of data. Third, machine learning could be fundamentally uninterpretable [10]. There is an inherent tension between machine learning performance (prediction accuracy) and interpretability. Generally, the best performing methods (such as deep learning) are the least interpretable, while the most interpretable methods (such as decision trees) are less accurate [11]. Therefore, some data-driven methods will not be used in fields with high safety coefficients, such as military and industrial fields. These fields require high precision and high efficiency. At present, the main methods of constructing interpretable models are as follows. First, an initial model is constructed using limited knowledge. Then, an optimization learning method is used to adjust the structure and parameters of the initial model. Through the above steps, the interpretability of the model can be guaranteed while improving its modeling [12]. To realize a trade-off between accuracy and interpretability, the belief rule base (BRB) (highly interpretable and accurate prediction) model is introduced in this paper.

Based on Dempster-Shafer's evidence theory [13], [14], decision theory [15], fuzzy theory [16], and traditional production rules [17], [18], Yang et al. proposed the BRB inference methodology by introducing the belief framework into traditional production rules in 2006 [19], [20]. BRB can effectively use quantitative and qualitative information to model a system. It has good interpretability [21], [22]. However, BRB has the problem of rule combination explosion when there are too many indicators in the data. An evidential reasoning (ER) algorithm is proposed for solving the BRB rule combination explosion problem in this paper. The ER algorithm is used to fuse the indicators and input the results into BRB to avoid the combinatorial explosion problem. In 1994, the ER method was first proposed by Yang and Singh and applied to the performance evaluation of motorcycles, which provides an effective way to solve multiattribute decision-making (MADM) problems [23]. The indicators are fused through the ER algorithm in the BRB execution process. Therefore, the ER algorithm parameters and BRB parameters are optimized at the same time, namely, by global optimization. This avoids the problem of local optimization. According to a literature query, this is the first time that a new EBRB-based model has been proposed to develop a UAV intrusion detection system.

Our main contributions are summarized as follows:

(1) This paper provides a method for solving the problem of BRB combination explosion in UAV intrusion detection. By introducing the ER algorithm, the combination explosion problem of the belief rule base is solved by ensuring the reasonable fusion of multiattribute indices.

(2) An interpretable global optimization detection model is constructed. A modeling process based on the EBRB model is constructed to provide an efficient and explicable reasoning process.

(3) To reduce the influence of the uncertainty of the initial parameters on the model detection accuracy, this paper uses the P-CMA-ES optimization algorithm to optimize the model parameters.

The remainder of this paper is organized as follows. In Section 2, the problem of UAV intrusion detection in a complex system is formulated and analyzed. In Section 3, a UAV intrusion detection model is constructed based on EBRB. An experimental case study is presented to verify the proposed model in Section 4. The conclusions of this study and future work are discussed in Section 5.

## 2 Problem formulation

Aiming at overcoming the problems that are encountered in the existing UAV intrusion detection systems, a new UAV intrusion detection model is proposed based on EBRB in this paper.

### 2.1 Problem formulation of UAV intrusion detection

Aiming at actual systems, the UAV intrusion detection model proposed in this paper will solve the following three problems:

(1) Combinatorial explosion of BRB

UAV intrusion detection and evaluation systems face many problems, such as many evaluation indices and complex systems. When using an ordinary BRB to build a model, the Cartesian product operation is required between indicators when building the initial BRB. Therefore, too many indicators will lead to the explosion of the BRB rule combination and affect the performance evaluation results [24]. To solve this problem, a hierarchical BRB model is often used. The use of a bottom-up model is the main strategy of a hierarchical BRB. First, the underlying indicators are combined. Then, the combination result is used as the input of the next layer. Finally, the process is terminated when the target state is reached [25]. The establishment of an evaluation system is the advantage of hierarchical BRBs according to the system structure. Compared with a single-layer BRB, the hierarchical structure of a hierarchical BRB effectively reduces the number of rules. Combinatorial explosion is effectively avoided. However, each layer is composed of several BRB models in a hierarchical BRB. Therefore, when the system is optimized, this model structure will lead to local optimization. This will affect the performance of the whole evaluation model. The sum of the local optima is not equal to the global optimum and may even be much smaller than the global optimum. To solve the local optimization and combinatorial explosion problems, an ER multiattribute fusion algorithm is proposed in this paper. First, ER is used for multiattribute fusion. Then, the fusion results are input into the BRB. The ER model and BRB model are integrated together. Therefore, the ER model is also optimized repeatedly when the BRB model is optimized repeatedly. Thus, the global optimization of the model is guaranteed. The fusion model is constructed using Equation (1):

(1) $$y(t)=ER(x_{1}(t),x_{2}(t),\cdots ,x_{J}(t),Q)$$

where y(t) is the fusion result of multiattribute data by the ER algorithm; ER(⋅) represents the fusion process of the ER algorithm; $x_{1}(t),x_{2}(t),\cdots ,x_{J}(t)$ are multiattribute data with *J* attributes; and *Q* is the parameter set of the ER algorithm for data fusion.

(2) Construction of an interpretable global optimization detection model

Due to the limitations of various application fields, such as military and other high-risk areas, traditional data-driven UAV intrusion detection models cannot be used. When making high-risk decisions according to an algorithm's results, it is important to know which functions have and have not been considered by the model. Artificial intelligence algorithms have always been “black boxes”, unable to provide a way to understand their internal processes. Therefore, these algorithms cannot be applied to some special fields. In addition, the relevant interpretable model is a local optimization process. This will lead to local optimization of the model. The construction of an interpretable global optimization model for UAV intrusion detection is the second problem to be solved. To solve this problem, a new model is proposed based on EBRB in this paper. Equation (2) is used to construct the intrusion detection model:

(2) $$u(S(y))=EBRB(y_{1}(t),y_{2}(t),\cdots ,y_{M}(t),V)$$

where u(⋅) denotes the result of UAV intrusion detection; S(⋅) is the UAV intrusion level; $y_{1}(t),y_{2}(t),\cdots ,y_{M}(t)$ means that the data fusion result is the input data of EBRB reasoning; *V* represents the set of parameters required by the EBRB reasoning process; and EBRB(⋅) represents the reasoning process of the model.

(3) Reduction of the influence of the uncertainty in the initial parameter values on the evaluation accuracy of the initial model

In the UAV intrusion detection model, the values of various parameters are difficult to accurately determine due to the complex mechanism of the UAV intrusion detection system. Therefore, the parameters need to be slightly adjusted to optimize the output results using an optimization algorithm. The basic strategy of optimization is to minimize the difference between the output of the prediction model and the output of the actual system. Therefore, optimization of the model parameters to reduce the impact of the uncertainty in the initial parameter values on the evaluation accuracy of the initial model is the third problem to be solved. To solve this problem, an optimization model based on P-CMA-ES is constructed. The optimization model is constructed using Equation (3):

(3) min⁡MSE=PCMAES(EBRB(⋅))

where min⁡MSE denotes minimization of the mean squared error value of the conjectured result and PCMAES(⋅) represents the optimization process of the EBRB model using the P-CMA-ES optimization algorithm.

### 2.2 Construction of a new UAV intrusion detection model

To solve the above three problems in engineering practice, a new UAV intrusion detection model based on EBRB is constructed in this subsection.

Based on EBRB, the structure of UAV intrusion detection model is as Equation (4):

(4) $$\begin{array}{c} R_{k}:\mathrm{IF}y_{1}\left(t\right)\;\mathrm{is}\;A_{1}^{k}\wedge y_{2}\left(t\right)\;\mathrm{is}\;A_{2}^{k}\wedge \cdots \wedge y_{M}\left(t\right)\;\mathrm{is}\;A_{M}^{k},\\ \mathrm{Then}\;S(t)\left\{(D_{1},\beta_{1,k}),\cdots ,(D_{N},\beta_{N,k})\right\}(\sum_{n=1}^{N}\beta_{n,k}\leq 1),\\ \text{With rule weight}\;\theta_{k}\;\text{and attribute weight}\;\delta_{1},\delta_{2},\cdots ,\delta_{M}\\ k\in \{1,2,\ldots ,L\} \end{array}$$

where $R_{k}\left(k=1,\cdots ,L\right)$ is the *kth* rule of the EBRB; *L* is defined as the number of rules in the EBRB; $y_{1}\left(t\right),y_{2}\left(t\right),\cdots ,y_{M}\left(t\right)$ is the input of the EBRB, namely, the ER algorithm fusion results; $\theta_{k}$ denotes the rule weight of the *kth* rule, reflecting the relative importance of the *kth* rule; $A_{i}^{k}$ is the reference value of the *ith* antecedent attribute; $D_{n}\left(j=1,\cdots ,N\right)$ is the evaluation level of the output; $\beta_{n,k}$ denotes the belief degree of $D_{n}$, where if $\sum_{n=1}^{N}\beta_{n,k}=1$, then the *kth* rule is complete, and otherwise, it is incomplete; and $\delta_{i}$ is the attribute weight of $y_{i}$, which represents the importance of the antecedent attribute. The structure of the UAV intrusion detection model is adjusted adaptively according to belief rules and parameters.

**Figure 1:**
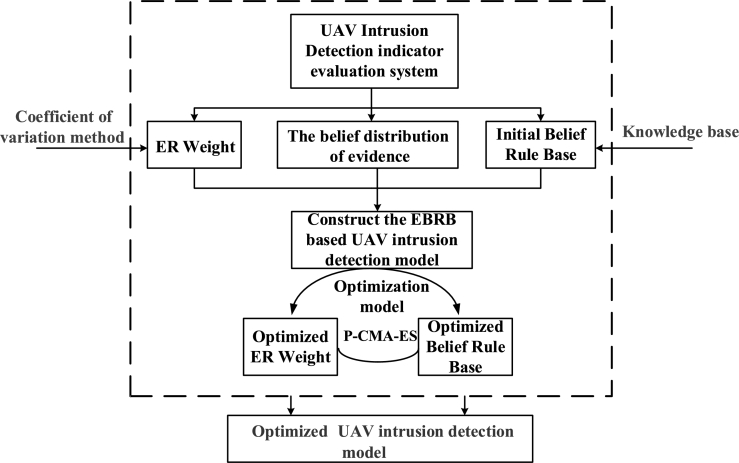
Modeling process of the UAV intrusion detection model.

For the developed UAV intrusion detection model, the modeling process considering both interpretability and global optimization is illustrated in Fig. 1.

## 3 UAV intrusion detection model based on EBRB

In this part, a new UAV intrusion detection model based on EBRB is developed. This model considers that BRBs have the disadvantage of rule combination explosion in the case of multiple attributes. The general method has the problem of local optimization. Therefore, a model structure based on EBRB is proposed. The structure of the intrusion detection model is adaptively adjusted based on the belief rules and weights, while maximizing its estimation accuracy is the optimization objective.

In Subsection 3.1, the reasoning process of the new intrusion detection model is constructed based on EBRB. In Subsection 3.2, the optimization process of the UAV intrusion detection model is constructed. The model structure of UAV intrusion detection is introduced in Subsection 3.3.

### 3.1 Reasoning process of the new UAV intrusion detection model

When different indicator data are used as the input of the intrusion detection model, they can be transformed into a unified attribute reference value matching degree through a reference value set by each indicator. The matching degree of the indicator input relative to the indicator reference value can be obtained by the following formula:

(5) $$a_{i}^{k}=\left\{ \begin{array}{cc} \frac{A_{i}^{l+1}-y_{i}\left(t\right)}{A_{i}^{l+1}-A_{i}^{l}}\; & k=l\left(A_{i}^{l}\leq y_{i}\left(t\right)\leq A_{i}^{l+1}\right)\\ \frac{y_{i}\left(t\right)-A_{i}^{l}}{A_{i}^{l+1}-A_{i}^{l}}\; & k=l+1\\ 0\; & k=1,2,\cdots ,K_{i}\left(k\neq l,l+1\right) \end{array}\right.$$

where $a_{i}^{k}$ is the matching degree of the input information with the *kth* rule; $y_{i}\left(t\right)$ denotes the *ith* antecedent attribute value in the input data, namely, the fusion results of the ER algorithm; $A_{i}^{l}$ and $A_{i}^{l+1}$ are defined as the reference values of the neighboring states of the *ith* antecedent attribute; and $K_{i}$ is the number of rules containing the *ith* indicator in the EBRB.

Having too many input indicators (too many $y_{i}\left(t\right)$) in the BRB will lead to the problem of rule combination explosion. To solve this problem, the ER algorithm is used as an indicator fusion method. The fusion result ($y_{i}\left(t\right)$) of the ER algorithm is used as the input of the BRB. By reasonably reducing the number of BRB input indicators, the problem of rule combination explosion is solved.

Suppose that the quantitative information input into the ER algorithm is $x_{i}$. The corresponding reference values are $h_{i,j}(i=1,\cdots ,L,j=1,\cdots ,J)$, where *J* represents the number of reference values. In this case, the decision-maker can establish a mapping relationship between the numerical value $x_{i,j}$ of $x_{i}$ and the reference value $h_{i,j}$. Based on the above discussion, Equation (6) is constructed:

(6) $$x_{i,j}meansh_{i,j}$$

Without loss of generality, it is assumed that decision-makers prefer reference value $h_{i,j+1}$ to reference value $h_{i,j}$. Let $h_{i,j}$ and $h_{i,j+1}$ be the maximum and minimum reference values, respectively. $x_{i}$ can be equivalently transformed into a belief distribution similar to that in Equation (7).

(7) $$e(x_{i})=\{(h_{i,j},p_{i,j}),i=1,\cdots ,L;j=1,\cdots ,J\}$$

The formula for $p_{i,j}$ is as follows:

(8) $$\left\{ \begin{array}{c} p_{i,j}=\frac{h_{i,j+1}-x_{i,j}}{h_{i,j+1}-h_{i,j}},h_{i,j}\leq x_{i,j}\leq h_{i,j+1},j=1,...,J-1\\ p_{i,j+1}=1-p_{i,j},h_{i,j}\leq x_{i,j}\leq h_{i,j+1},j=1,...,J-1\\ p_{i,k}=0,k=1,...,J;k\neq j,j+1 \end{array}\right.$$

Based on the standardized indicator data in Equation (8) and the indicator weight determined by the coefficient of variation method [26], the ER algorithm is used to fuse indicator data and parameters. According to the calculation formula of the ER algorithm, the implementation process is analyzed in detail. The weight of evidence is $q_{i}(i=1,...,I)$, which satisfies $0\leq q_{i}\leq 1$. The ER algorithm can be expressed by the following Equation (9):

(9) $$\psi_{n}=\frac{\upsilon [\prod_{k=1}^{L}(q_{k}p_{n,k}+1-q_{k}\sum_{j=1}^{N}p_{j,k})-\prod_{k=1}^{L}(1-q_{k}\sum_{j=1}^{N}p_{j,k})]}{1-\upsilon [\prod_{k=1}^{L}(1-q_{k})]}\;\upsilon ={\left[\sum_{n=1}^{N}\prod_{k=1}^{L}(q_{k}p_{n,k}+1-q_{k}\sum_{j=1}^{N}p_{j,k})-(N-1)\prod_{k=1}^{L}(1-q_{k}\sum_{j=1}^{N}p_{j,k})\right]}^{-1}$$

where $\psi_{n}$ is the belief level of the *nth* output result grade $H_{n}$ obtained by fusing the input index monitoring data, $0\leq p_{n}\leq 1$, $\sum_{n=1}^{N}p_{n}=1$, and $p_{j,k}$ represents the basic belief degree of the *jth* reference level of the output of the *kth* rule.

Suppose the utility of evaluation grade $H_{n}$ is $u(H_{n})$. The expected utility of the evaluation scheme is calculated by the utility-based method. As shown in Equation (10):

(10) $$y(t)=\sum_{n=1}^{N}u(H_{n})\psi_{n}$$

where y(t) denotes the data fusion results of UAV intrusion detection. y(t) is the input of the BRB, namely, the input in Equation (5).

After the matching degree is obtained through Equation (5), the activation weight is calculated, namely, the activation degree of the information input into the rule. The activation weight is calculated as Equation (11):

(11) $$w_{k}=\frac{\theta_{k}\prod_{i=1}^{M}{\left(a_{i}^{k}\right)}^{\delta_{i}}}{\sum_{l=1}^{K}\theta_{l}\prod_{i=1}^{M}{\left(a_{i}^{l}\right)}^{\delta_{i}}}$$

where $w_{k}$ is the activation weight of the *kth* rule; $\theta_{k}$ denotes the rule weight of the *kth* rule; $\delta_{i}$ represents the *ith* antecedent attribute weight; and *M* is the number of antecedent attributes.

After calculating the activation weights of the belief rules, they can be combined by the ER algorithm. The algorithm is as Equation (12):

(12) $$\beta_{n}=\frac{\mu [\prod_{k=1}^{L}(w_{k}\beta_{n,k}+1-w_{k}\sum_{j=1}^{N}\beta_{j,k})-\prod_{k=1}^{L}(1-w_{k}\sum_{j=1}^{N}\beta_{j,k})]}{1-\mu [\prod_{k=1}^{L}(1-w_{k})]}\;\mu ={\left[\sum_{n=1}^{N}\prod_{k=1}^{L}(w_{k}\beta_{n,k}+1-w_{k}\sum_{j=1}^{N}\beta_{j,k})-(N-1)\prod_{k=1}^{L}(1-w_{k}\sum_{j=1}^{N}\beta_{j,k})\right]}^{-1}$$

After fusing the input indicator data, $\beta_{n}$ is the belief degree of the *nth* obtained output result level $D_{n}$, which satisfies $0\leq \beta_{n}\leq 1$ and $\sum_{n=1}^{N}\beta_{n}=1$. $\beta_{j,k}$ represents the belief degree of the *jth* reference level in the output of the *kth* rule.

After merging the rules, the final output result of the EBRB model can be expressed as Equation (13):

(13) $$S(y_{i})=\{(D_{n},\beta_{n});n=1,2,\ldots ,N\}$$

where $y_{i}$ is the input data of the *ith* indicator and *S* is the UAV intrusion level. For the *nth* result level $D_{n}$, the evaluation utility can be expressed as $u(D_{n})$. After the activated rules are fused, the final UAV intrusion detection result can be obtained:

(14) $$\overset{ˆ}{u}(S(y))=\sum_{n=1}^{N}u(D_{n})\beta_{n}$$

where $\overset{ˆ}{u}(S(y))$ is the final output result of the UAV intrusion detection as Equation (14).

### 3.2 Optimization process of the UAV intrusion detection model

To reduce the impact of initial parameter uncertainty on the evaluation model, the parameters of the model need to be adjusted in combination with the collected data. By adjusting the parameters, the evaluation accuracy of the model for UAV intrusion detection is improved. In this model, the model accuracy is the objective function of optimization, which is expressed as the MSE of the performance state of the actual system and the output of the model. As shown in Equation (15), the MSE is constructed.

(15) $$MSE(ℜ)=\frac{1}{T}\sum_{t=1}^{T}{\left(\overset{ˆ}{u}(t)-u(t)\right)}^{2}$$

where $\overset{ˆ}{u}(t)$ represents the output value of the model, namely, the size of the error coefficient; u(t) represents the true value of the output; *T* is the number of model input data; and ℜ is the vector of parameters to be optimized.

The constraints of the parameters are as follows in the EBRB model:

(16) $$\begin{array}{c} \min MSE(EBRB(ℜ))\\ \left\{ \begin{array}{c} 0\leq q_{j}\leq 1,j=1,2,\ldots ,J\\ 0\leq \theta_{k}\leq 1,k=1,2,\cdots ,L\\ 0\leq \delta_{i}\leq 1,i=1,2,\cdots ,M\\ 0\leq \beta_{n,k}\leq 1,n=1,2,\cdots ,N;k=1,2,\cdots L\\ \sum_{j}^{J}q_{j}=1,j=1,2,\ldots J\\ \sum_{n=1}^{N}\beta_{n,k}\leq 1,k=1,2,\cdots L \end{array}\right. \end{array}$$

where ℜ=[q,θ,δ,β] represents the parameter set of the EBRB model.

In this paper, the CMA-ES algorithm is used as the optimization function. The full name of CMA-ES is the covariance matrix adaptation evolution strategy [27], [28]. It is one of the most important optimization algorithms. It performs well on high-dimensional nonlinear optimization problems and can quickly converge to the global optimum with fewer individuals [29], [30].

The CMA-ES algorithm can be used to solve nonlinear and nonconvex real-valued continuous optimization problems [28]. It controls the evolutionary direction of the whole population by adjusting the covariance matrix. A small-scale population can quickly converge to the optimal solution. The CMA-ES algorithm is mainly composed of three parts: sampling, selection and reorganization, and updating the covariance matrix. However, the CMA-ES algorithm is only suitable for solving unconstrained optimization problems or boundary-constrained problems. The parameters are constrained in this paper. The parameters need to be optimized. Therefore, it needs to be improved into a constrained optimization algorithm.

The P-CMA-ES algorithm with a projection operation is used in this paper. The projection operation is used to directly map a solution that does not satisfy the constraint back to the feasible region, so that it satisfies the constraint. In addition, the P-CMA-ES algorithm has the same time and space complexity as the original algorithm. The projection operation is used to solve the equality constraints in the above objective function (Equation (16)).

Remark 1 The EBRB model proposed in this paper is a global optimization model. The parameters in the ER algorithm and BRB are mixed and optimized under the constraints of the objective function. Under the limitation of the optimization objective function, the ER algorithm is executed repeatedly under the condition of repeated execution of the BRB. Suppose the number of optimization rounds is set to 100. In the 56th optimization round, the number of offspring in the optimization algorithm is lambda=10+floor(3⁎size(x)). Then, the BRB algorithm is executed lambda/2 times. In each optimization round, the parameters from the last optimization round are applied to the next optimization round until lambda/2 optimization rounds are completed. Then, the 57th optimization round is carried out, and the above process is repeated. The number of executions of the ER algorithm is size(e)/size(x)⁎lambda.

**Figure 2:**
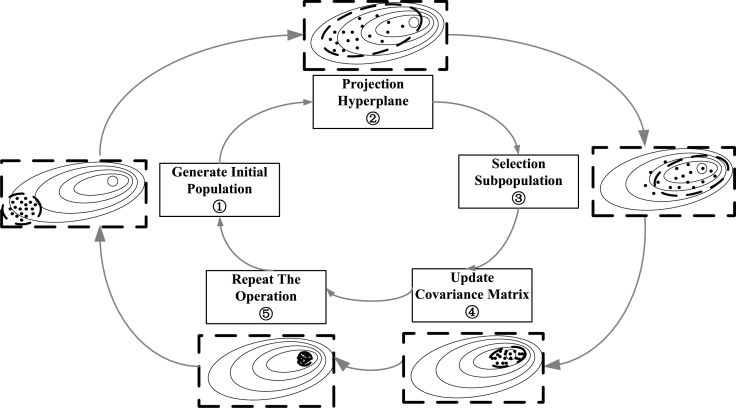
Optimization process of P-CMA-ES.

As illustrated in Fig. 2, the optimization process of P-CMA-ES in this model can be completed through the following steps:

Step 1: Sampling operation. Taking the initial solution as the center (expected value), a multidimensional ellipsoid population (a population with a normal distribution) is generated through a normal distribution.

Step 2: Projection operation. The solution is projected onto a hyperplane. The parameters are constrained.

Step 3: Selection and reorganization operations. The population that satisfies the constraints and approaches the optimal solution is selected as the subpopulation.

Step 4: Updating the covariance matrix. When all solutions in the population meet the constraints, the covariance matrix of the population is updated.

Step 5: Repeating the above operations. Until the accuracy requirements are met, the final optimal parameter ${ℜ}_{best}$ is output.

**Figure 3:**
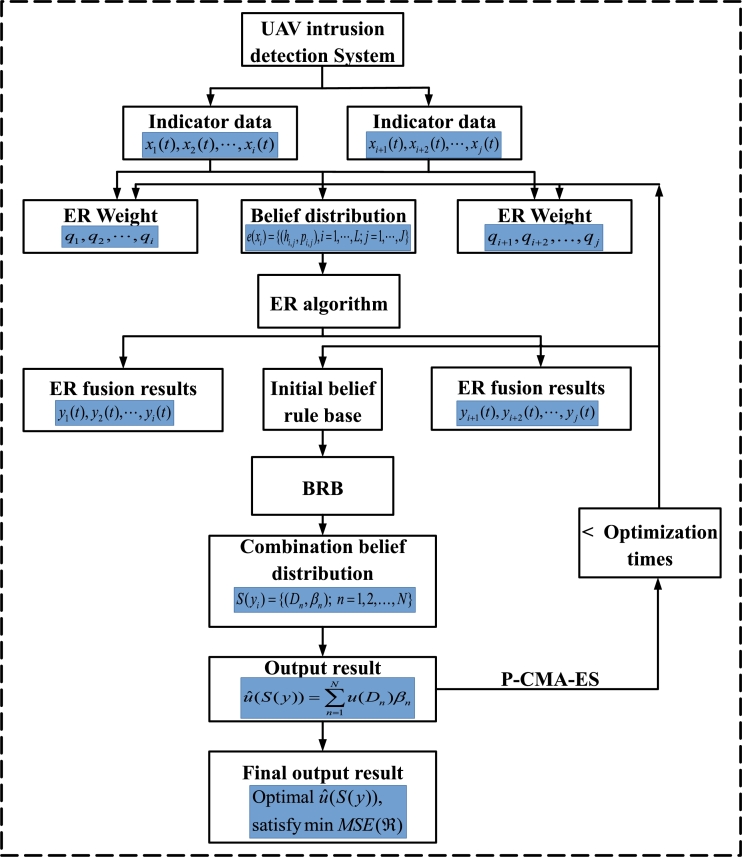
UAV intrusion detection execution process.

The execution process of UAV intrusion detection based on the EBRB model is illustrated in Fig. 3.

### 3.3 Model structure of UAV intrusion detection

**Figure 4:**
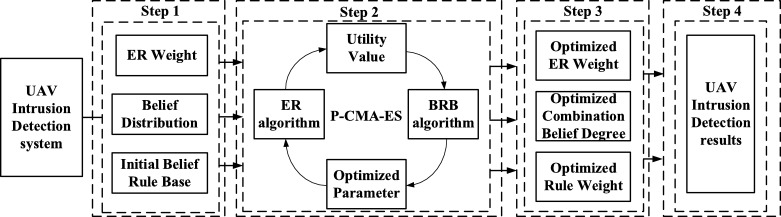
The structure of the UAV intrusion detection model.

As illustrated in Fig. 4, the structure of the UAV intrusion detection model can be described by the following steps.

Step 1: Construction of the UAV intrusion detection evaluation model. The weight is obtained by the coefficient of variation method. The belief distribution of evidence is obtained by a rule-based method. Initial belief rules are constructed.

Step 2: Construction of the intrusion detection optimization model based on EBRB. The P-CMA-ES optimization algorithm is used to optimize the model parameters.

Step 3: Obtaining the optimized model parameters. Based on the optimized model, the test data are input into the model.

Step 4: Testing of the optimized UAV intrusion detection model on the testing data. Finally, the intrusion detection evaluation results are obtained.

## 4 Case study

In this section, a UAV intrusion detection dataset [31] is used to demonstrate the implementation and validity of the proposed EBRB model. To avoid the failure of existing physical detection methods (such as radar, vision and sound) in many cases, UAV-encrypted Wi-Fi traffic data records can be a very promising source for detecting UAV intruders. For the UAV intruder detection dataset, each input is an encrypted Wi-Fi traffic record, while the output is whether the current traffic is from a UAV or not. If the traffic is from a UAV, it indicates that the area has been invaded by the UAV. On this dataset, there are two different types of models: a bidirectional-flow model and a unidirectional-flow model. Without loss of generality, the unidirectional-flow pattern of Parrot Bebop is considered. In addition, there are 18 indicators in this model.

This case includes three parts. The UAV intrusion detection evaluation model is constructed in Subsection 4.1. In Subsection 4.2, the training and testing of the UAV intrusion detection model are presented. Comparative experiments are discussed in Subsection 4.3.

### 4.1 Construction of the UAV intrusion detection evaluation model

Based on the UAV intrusion detection evaluation method developed in Section 3, the intrusion detection model is constructed combined with the obtained indicator data.

The dataset has the two key attributes: packet size and packet arrival interval. From these two key attributes, 9 relevant characteristics are obtained through statistical analysis. According to the statistical collection process of the dataset, the indicator of the dataset is divided into a packet size ($\xi_{1}$)-related indicator of influencing factors, namely, ($x_{1}-x_{9}$), and a packet arrival interval ($\xi_{2}$)-related indicator of influencing factors, namely, ($x_{10}-x_{18}$).

Combined with UAV-related information and evidence reasoning knowledge, the reference points and reference values of $\xi_{1}$ and $\xi_{2}$ are obtained. The weight of the indicator is obtained by the coefficient of variation method.

There are 4 reference points for the indicators in $\xi_{1}$, namely, small (S), middle (M), large (L), and very large (VL).

There are 5 reference points for the indicators in $\xi_{2}$, namely, small (S), a little small (LS), middle (M), large (L), and very large (VL).

Using a rule-based method, such as that expressed by Equation (8), the data are transformed by consistency transformation into a belief distribution, such as that expressed by Equation (7). The following is an example of a belief transformation: Suppose the value of indicator $x_{1}$ is 0.00041, $p_{1,1}=0$, $p_{1,2}=\frac{0.002741-0.00041}{0.002741-0.0002635}=0.9409$, $p_{1,3}=1-\frac{0.002741-0.00041}{0.002741-0.0002635}=0.0591$, $p_{1,4}=0$. Therefore, the belief distribution of Equation (7) can be expressed as:

(17) e(x)={(S,0),(M,0.9409),(L,0.0591),(VL,0)}

as presented in Table 1 and Table 2.

**Table 1:** Reference points, reference values and weights of *ξ*_1_-related indicators.

Indicator	Reference point	Reference value	Weight
*x*_1_	(S, M, L, VL)	(8.7000×10^−5^, 0.0002635, 0.002741, 36.1423)	0.1213
*x*_2_	(S, M, L, VL)	(5.6900×10^−5^, 0.0003492, 0.006328, 342.2272)	0.1499
*x*_3_	(S, M, L, VL)	(1.0000×10^−6^, 0.0001155, 0.00035, 0.8174)	0.1349
*x*_4_	(S, M, L, VL)	(1.6900×10^−13^, 9.04×10^−5^, 0.0001586, 0.5551)	0.1507
*x*_5_	(S, M, L, VL)	(−0.3775, 1.806, 4.735, 9.7020)	0.0166
*x*_6_	(S, M, L, VL)	(−1.9807, 1.69, 8.917, 93.0596)	0.0313
*x*_7_	(S, M, L, VL)	(3.2700×10^−4^, 0.002944, 0.08582, 3.4232×10^3^)	0.1777
*x*_8_	(S, M, L, VL)	(0, 0.000108, 0.000178, 0.0054)	0.0708
*x*_9_	(S, M, L, VL)	(1.3694×10^−4^, 0.0007815, 0.01213, 342.4245)	0.1469

**Table 2:** Reference points, reference values and weights of *ξ*_2_-related indicators.

Indicator	Reference point	Reference value	Weight
*x*_10_	(S, LS, M, L, VL)	(65, 87.37, 104.6, 1393, 1.6681×10^3^)	0.0859
*x*_11_	(S, LS, M, L, VL)	(0.5628, 33.91, 35.92, 37.7, 694.2856)	0.0811
*x*_12_	(S, LS, M, L, VL)	(62, 138, 1476, 1596, 1676)	0.0945
*x*_13_	(S, LS, M, L, VL)	(0, 20.76, 47.44, 105.3, 1.0022×10^3^)	0.2499
*x*_14_	(S, LS, M, L, VL)	(−9.7020, −0.8598, 0.03941, 0.7602, 9.7020)	0.0209
*x*_15_	(S, LS, M, L, VL)	(−2.0199, −1.836, −1.436, −0.6021, 93.0597)	0.1950
*x*_16_	(S, LS, M, L, VL)	(68, 138, 153, 1476, 1676)	0.0724
*x*_17_	(S, LS, M, L, VL)	(62, 222, 288, 320, 1632)	0.1181
*x*_18_	(S, LS, M, L, VL)	(65.0692, 95.73, 112.5, 1446, 1.6693×10^3^)	0.0820

In this model, $S=\left\{S_{1},S_{2}\right\}$ is used to represent the intrusion level of the UAV. $y_{1}$ is used to represent the related attribute results of $\xi_{1}$ attributes fused by ER. $y_{2}$ is used to represent the related attribute results of $\xi_{2}$ attributes fused by ER. Combined with the obtained results, the reference levels and reference values presented in Table 3 and Table 4 are determined. $y_{1}$ is described by using four levels of semantic value: “very unlikely intrusion (VU)”, “possible intrusion (PI)”, “very likely intrusion (VL)”, and “basically sure intrusion (BI)”. $y_{2}$ is described by using five levels of semantic value: “very unlikely intrusion (VU)”, “not too possible intrusion (NI)” “possible intrusion (PI)”, “very likely intrusion (VL)”, and “basically sure intrusion (BI)”. The resulting attribute a is set to 2 reference points, which are nonintrusion (NI) and intrusion (I), whose reference values are 0 and 1, respectively. According to the belief rule construction process, a total of 20 belief rules are generated for four states of $y_{1}$ and five states of $y_{2}$. Additionally, the initial UAV intrusion detection model is constructed by randomly assigning the belief degrees of the output results in the rules.

**Table 3:** Reference points and reference values for *y*_1_.

Reference point	VU	PI	VL	BI
Reference value	1	2	3	4

**Table 4:** Reference points and reference values for *y*_2_.

Reference point	VU	NI	PI	VL	BI
Reference value	1	2	3	4	5

### 4.2 UAV intrusion detection model training and testing

After constructing the model, the parameters need to be adjusted due to uncertainty. Therefore, it is necessary to use the training data to adjust and modify the parameters of the model for evaluation. Through parameter adjustment, the model evaluation accuracy is improved for UAV intrusion detection.

In this subsection, the UAV intrusion detection model is trained based on the acquired data. In the training part of this model, there are 80 training parameters. The training parameters are the indicator weights, rule output belief degrees, and rule weights. A total of 1062 groups of monitoring data are used in the experiment. In the experimental part, 743 groups are randomly selected as training data, and the remaining 319 groups are selected as test data. Based on the UAV intrusion detection evaluation model constructed in Section 3, P-CMA-ES is used to adjust and optimize the model parameters. In addition, the number of training iterations of the optimization model is set to 200.

**Figure 5:**
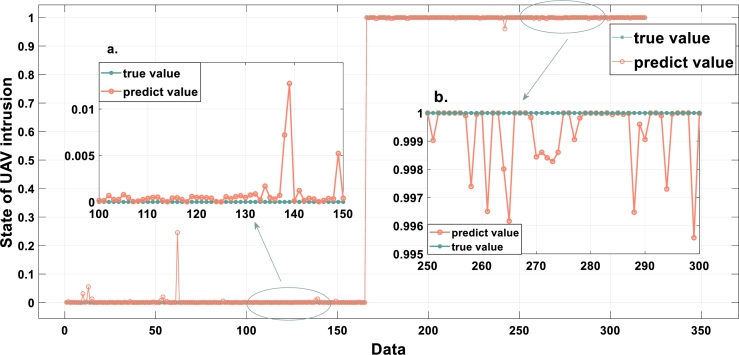
Fitting diagram of the EBRB model in this paper. a. Enlarged view of 100-150 data b. Enlarged view of 250-300 data.

**Figure 6:**
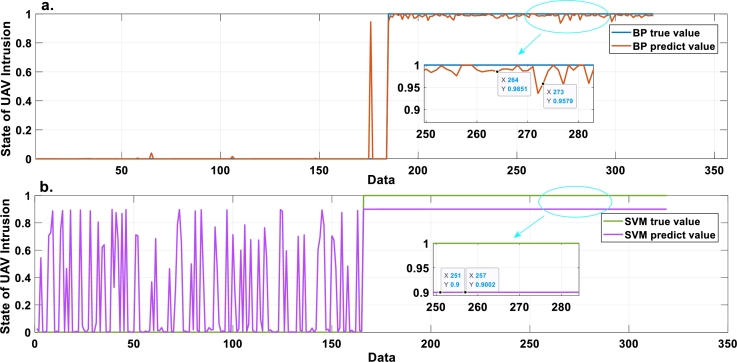
Fitting diagram of compared models. a. BP model b. SVM model.

After training, the optimized UAV intrusion detection evaluation model and weights are presented in Table 5, Table 6, and Table 7. The trained model is used for testing. The model test results and the actual results of UAV intrusion are compared in Fig. 5. The MSE of the model output is $2.1033\times 10^{-4}$. As shown in Fig. 5, the model can accurately distinguish the UAV intrusion state, where the true value is the actual state of UAV intrusion and the predicted value is the output result of this model. An output diagram of the comparison model is shown in Fig. 6.

**Table 5:** UAV intrusion detection evaluation model after training.

Serial number	Rule weight	*y*_1_∧*y*_2_	UAV intrusion levels {*S*_1_,*S*_2_}
1	0.0084	VU ∧ VU	{0.1083, 0.8917}
2	0.3748	VU ∧ NI	{0.0026, 0.9974}
3	0.1521	VU ∧ PI	{0.0095, 0.9905}
4	0.8074	VU ∧ VL	{0.5738, 0.4262}
5	0.6856	VU ∧ BI	{0.4273, 0.5727}
6	0.0049	PI ∧ VU	{0.4266, 0.5734}
7	0.7136	PI ∧ NI	{0, 1}
8	0.0016	PI ∧ PI	{0.3115, 0.6885}
9	0.3057	PI ∧ VL	{0.9737, 0.0263}
10	0.1329	PI ∧ BI	{0.6419, 0.3581}
11	0.1446	VL ∧ VU	{0.2982, 0.7018}
12	0.0037	VL ∧ NI	{0.5369, 0.4631}
13	0.9308	VL ∧ PI	{1, 0}
14	0.9267	VL ∧ VL	{0.9993, 0.0007}
15	0.0376	VL ∧ BI	{0.6474 0.3526}
16	0.9855	BI ∧ VU	{0.2244, 0.7756}
17	0.9368	BI ∧ NI	{0.8241, 0.1759}
18	0.2863	BI ∧ PI	{0.0550, 0.9450}
19	0.1463	BI ∧ VL	{0.4359, 0.5641}
20	0.0653	BI ∧ BI	{0.3271, 0.6729}

**Table 6:** Optimized weights of relevant indicators in *ξ*_1_.

*x*_1_	*x*_2_	*x*_3_	*x*_4_	*x*_5_	*x*_6_	*x*_7_	*x*_8_	*x*_9_
0.7502	0.3592	0.9457	0.2276	0.2937	0.0565	0.7188	0.1814	0.2952

**Table 7:** Optimized weights of relevant indicators in *ξ*_2_.

*x*_10_	*x*_11_	*x*_12_	*x*_13_	*x*_14_	*x*_15_	*x*_16_	*x*_17_	*x*_18_
0.5670	0.1277	0.1068	0.1435	0.1141	0.3937	0.2987	0.3440	0.0835

### 4.3 Comparative experiment on the UAV intrusion detection evaluation model

To evaluate the traffic flow prediction performance of the RNN-GCN model, this paper introduces three evaluation indicators for measuring the prediction performance of the model, where u(t) represents the actual result at time *t*, $\overset{ˆ}{u}(t)$ represents the forecast data output by the model at the first time, and *n* is the total number of test samples:

(i) Mean squared error (MSE)

(18) $$MSE=\frac{1}{n}\sum_{i=1}^{n}{\left(\overset{ˆ}{u}(t)-u(t)\right)}^{2}$$

*MSE* can be used to evaluate the prediction accuracy of the model. The smaller the MSE value is, the more accurate the fit of the prediction model to the target.

(ii) Root mean square error (RMSE)

(19) $$RMSE=\sqrt{\frac{1}{n}\sum_{i=1}^{n}{\left(\overset{ˆ}{u}(t)-u(t)\right)}^{2}}$$

RMSE is used to measure the deviation between the predicted value and the true value and is more sensitive to outliers in the data. The smaller the value is, the better the prediction performance is.

(iii) Mean absolute error (MAE)

(20) $$MAE=\frac{1}{n}\sum_{i=1}^{n}\left|\overset{ˆ}{u}(t)-u(t)\right|$$

*MAE* can well reflect the actual situation of the predicted value error and is the first choice for model prediction performance evaluation. The smaller the value is, the better the prediction performance is.

**Figure 7:**
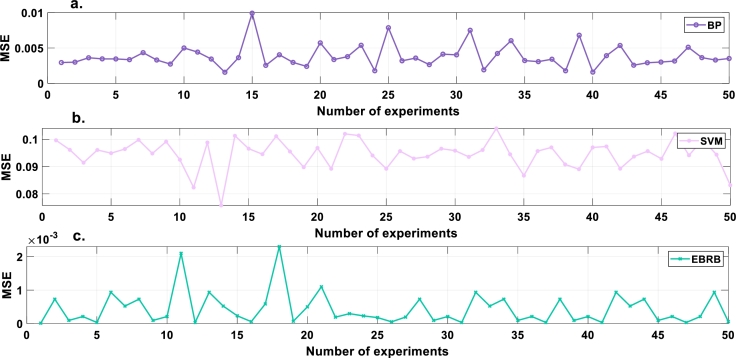
MSE values of compared models. a. BP b. SVM c. EBRB.

**Figure 8:**
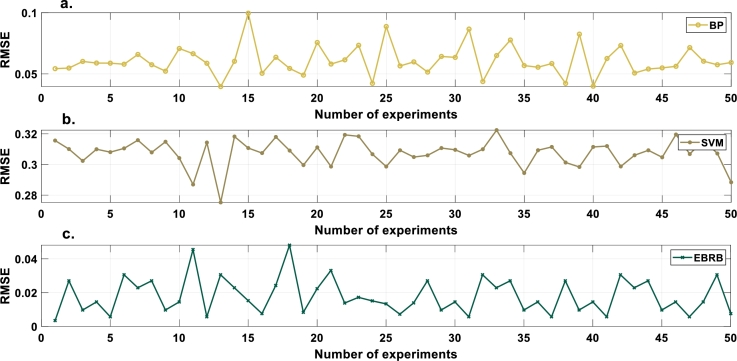
RMSE values of compared models. a. BP b. SVM c. EBRB.

**Figure 9:**
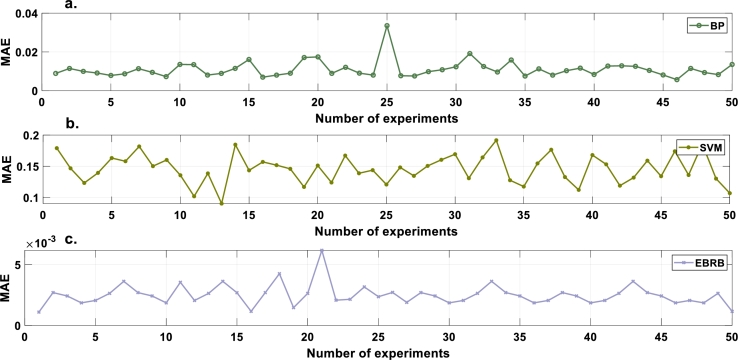
MAE values of compared models. a. BP b. SVM c. EBRB.

To prove the excellent robustness of the optimization model, the experiment is repeated 50 times. After 50 experimental repetitions, the average MSE, RMSE and MAE values are 4.3075×10^−4^, 1.7937×10^−2^ and 2.5019×10^−3^, respectively. To evaluate the performance of the intrusion detection model constructed in this paper, a backpropagation neural network (BP) and SVM are used for comparative experiments. The average results of 50 repeated experiments are presented in Table 8. According to Table 8, the UAV intrusion detection model is proven to have good robustness in this paper. Figure 7, Figure 8, Figure 9 show the MSE, RMSE and MAE values, respectively, of the repeated model experiments.

**Table 8:** Comparison of MSE, RMSE and MAE values.

Model	EBRB	BP	SVM
Average MSE	4.3075×10^−4^	3.8332×10^−3^	9.4590×10^−2^
Average RMSE	1.7937×10^−2^	6.0724×10^−2^	3.0743×10^−1^
Average MAE	2.5019×10^−3^	1.1061×10^−2^	1.4566×10^−1^

To further prove the robustness of the model, the accuracy and precision of the model are further evaluated when the number of training data is gradually reduced. Moreover, our model is compared with KNN, logistic regression (LR), NB, SVM, decision tree (DT) and random forest (RF). The model experiment is implemented in Python and MATLAB. The experiment is repeated 50 times for each model. The average accuracy, precision and F1_score results are presented in Table 9.

**Table 9:** Comparison of accuracy, precision and F1_score.

Model	Training: 10%, Test: 90%			Training: 30%, Test: 70%		
	Accuracy	Precision	F1_score	Accuracy	Precision	F1_score
KNN	89.85%	81.87%	90.03%	92.34%	86.06%	92.51%
LR	95.40%	91.74%	95.16%	96.77%	96.86%	96.58%
NB	96.03%	92.02%	95.84%	95.30%	90.96%	95.26%
SVM	90.59%	82.95%	90.68%	92.61%	86.49%	92.75%
DT	96.05%	92.12%	95.88%	92.57%	86.46%	92.73%
RF	98.22%	98.29%	98.22%	98.79%	98.82%	98.79%
EBRB	99.60%	99.64%	99.55%	99.82%	99.81%	99.81%



Model	Training: 50%, Test: 50%			Training: 70%, Test: 30%		
	Accuracy	Precision	F1_score	Accuracy	Precision	F1_score
KNN	95.10%	90.51%	95.02%	98.75%	97.47%	98.72%
LR	97.55%	96.44%	97.41%	98.43%	96.86%	98.40%
NB	95.29%	90.84%	95.20%	97.81%	95.88%	97.90%
SVM	93.60%	87.94%	93.58%	93.73%	88.51%	93.90%
DT	95.54%	91.29%	95.44%	96.14%	92.62%	96.17%
RF	98.87%	98.90%	98.87%	97.81%	95.65%	97.78%
EBRB	99.94%	99.90%	99.94%	99.95%	99.90%	99.95%

With the gradual reduction of the size of the training set, the indicator values of the proposed method remain above 99%, as presented in Table 9. An interpretable model should be considered for UAV intrusion detection because such a model is more structured, causal, and compact. Research shows that good performance may be realized by using learning models such as KNN, SVM and RF. However, their interpretability is not strong. Although NB, LR and DT have some interpretability, their performances are not as good as that of the method proposed in this paper. The interpretable UAV intrusion detection evaluation method proposed in this paper focuses on improving the interpretation ability. Moreover, our model also maintains a high level of learning performance compared to a series of machine learning technologies. Table 10 presents the hyperparameters of the compared models.

**Table 10:** Comparison Model hyperparameters.

	Training: 10%, Test: 90%	Training: 30%, Test: 70%
KNN	n_neighbors=68, weights= ‘uniform’, algorithm= ‘auto’, leaf_size=30, p=2, metric= ‘minkowski’, metric_params=None, n_jobs=1	n_neighbors=158, weights= ‘uniform’, algorithm= ‘auto’, leaf_size=30, p=2, metric= ‘minkowski’, metric_params=None, n_jobs=1
LR	C = 0.0071	C = 0.00101
NB	var_smoothing = 0.033	var_smoothing = 0.06
SVM	kernel=“poly”, C=0.056, probability=True	kernel=”poly”, C=0.025, probability=True
DT	min_samples_leaf = 53	min_samples_leaf = 158
RF	n_estimators=8, oob_score=True, n_jobs=1, random_state=101, max_features=None, min_samples_leaf=34	n_estimators=70, oob_score=True, n_jobs=1, random_state=101, max_features=None, min_samples_leaf=99



	Training: 50%, Test: 50%	Training: 70%, Test: 30%
KNN	n_neighbors=80, weights= ‘uniform’, algorithm= ‘auto’, leaf_size=30, p=2, metric= ‘minkowski’, metric_params=None, n_jobs=1	n_neighbors=50, weights= ‘uniform’, algorithm= ‘auto’, leaf_size=30, p=2, metric= ‘minkowski’, metric_params=None, n_jobs=1
LR	C = 0.00052	C = 0.00051
NB	var_smoothing = 0.057	var_smoothing = 0.008
SVM	kernel=“poly”, C=0.025, probability=True	kernel=”poly”, C=0.025, probability=True
DT	min_samples_leaf = 262	min_samples_leaf = 360
RF	n_estimators=70, oob_score=True, n_jobs=1, random_state=101, max_features=None, min_samples_leaf=166	n_estimators=70, oob_score=True, n_jobs=1, random_state=101, max_features=None, min_samples_leaf=225

## 5 Conclusions

UAV intrusion detection has attracted increasing attention in various fields. Aiming to solve the security and accuracy problems in the process of UAV intrusion detection, an EBRB model was proposed in this paper.

A new interpretable global optimization method was proposed for UAV intrusion detection in this paper. The feasibility of the method was verified by experiments, which has potential engineering application value. In future work, we will continue to study the security deduction of UAV intrusion detection. Security deduction determines the cause of intrusion and detects the intrusion of UAVs through reverse security reasoning.

Through research on the causes of intrusion, the UAV intrusion detection model will be further strengthened. Through continuous development, a spiral principal UAV intrusion detection system will be formed, and the detection ability of the system will be continuously improved.

## Declarations

### Author contribution statement

Yawen Xie: Conceived and designed the experiments; Performed the experiments; Analyzed and interpreted the data; Contributed reagents, materials, analysis tools or data; Wrote the paper.

Wei He, Hailong Zhu, Ruohan Yang, Quanqi Mu: Conceived and designed the experiments; Analyzed and interpreted the data.

### Funding statement

Dr Wei He was supported by the Postdoctoral Science Foundation of China [2020M683736], the 10.13039/501100005046Natural Science Foundation of Heilongjiang Province of China [LH2021F038], the innovation practice project of college students in Heilongjiang Province [202010231009, 202110231024, 202110231155], the graduate quality training and improvement project of 10.13039/501100013772Harbin Normal University [1504120015], the graduate academic innovation project of 10.13039/501100013772Harbin Normal University [HSDSSCX2021-120, HSDSSCX2021-29].

### Data availability statement

Data associated with this study has been deposited at http://mason.gmu.edu/~lzhao9/materials/data/UAV/

### Declaration of interests statement

The authors declare no conflict of interest.

### Additional information

No additional information is available for this paper.
